# Dataset of lithium-ion battery degradation based on a forklift mission profile for state-of-health estimation and lifetime prediction

**DOI:** 10.1016/j.dib.2023.109861

**Published:** 2023-11-27

**Authors:** Søren B. Vilsen, Daniel-Ioan Stroe

**Affiliations:** a Department of Mathematical Sciences, Aalborg University, Aalborg East, 9220, Denmark; b Daniel-Ioan Stroe: Department of Energy, Aalborg University, Aalborg East, 9220, Denmark

**Keywords:** Battery, Lithium-ion, Degradation, Cycle ageing, Forklift operation

## Abstract

Lithium-ion (Li-ion) batteries are becoming an increasingly integral part of modern society, through consumer electronics, stabilisation of the electric grid, and electric vehicles. However, Lithium-ion batteries degrade in effectiveness over time; a degradation which is extremely dependent on the usage of the battery. Therefore, to study how a battery cell degrades under dynamic conditions, a realistic load profile was constructed based on the operation of forklifts. This profile was used to age three Lithium-ion battery cells at 45, 40, and 35*°*C and the response of the cells was measured on a second-by-second basis. Periodically the ageing was halted to perform a reference test of the cells allowing for the tracking of their degradation.

Specifications Table

**Table:** 

Subject	Energy, Batteries, Electrical Engineering
Specific subject area	Lithium-ion battery degradation using a forklift operation profile.
Data format	Raw
Type of data	Raw second-by-second measurement data stored as .csv files.
Data collection	The data were collected by performing ageing experiments using a realistic forklift load profile at three temperatures. The devices under test are prismatic Lithium-ion batteries with a nominal capacity of 180 Ah and a nominal voltage of 3.2 V. The ageing was performed using a Digatron battery test station MCT 50-06-24. During all the experiments, the battery cells were placed in climatic chambers to ensure reliable and uniform temperature distribution. At the end of each experiment, the generated data was saved using the Digatron Battery Manager Workstation and stored as .csv files.
Data source location	Department of Energy, Aalborg University, Aalborg East, Denmark.
Data accessibility	Repository name: Mendeley data Data identification number: 10.17632/yz4pttm73n.2 Direct URL to data: https://data.mendeley.com/datasets/yz4pttm73n

## 1 Value of the Data

• This dataset contains high-frequency measurements (at the resolution of 1s) of three battery cells which were aged using a realistic forklift load profile at three elevated temperatures, respectively. These measurements were recorded both during battery ageing (i.e., forklift operation) and reference performance testing (RPT), giving insight and access to the performance-degradation behaviour of the battery cells when aged with the profile at different temperatures, following the procedure in [1].
• The operation profile used to age the battery cells, is based on the real field operation of forklifts. While this could be a weakness, as forklifts are a niche application, it can also be a strength, because the operations is very similar to that of an electric vehicle (i.e., dynamic discharge imposed by the driver followed by partial charging). Thus, this operation profile offers an alternative both for modelling and validating the generalization capability of methods and algorithms developed for electric vehicles.
• Due to the high frequency of the measurements and longevity of the study, this dataset will be useful for researchers interested in both short- and long-term battery state-of-charge (SOC) and state-of-health (SOH) modelling, estimation, and prediction.
• As this dataset contains both operation and reference performance measurements, it is ideal for researchers interested in the interplay between the health and operation of the battery cell (i.e., the effect of the operation on the battery performance-degradation behaviour, and vice versa).
• The battery cells were aged under realistic operating conditions, thus, these data provide a solid foundation for training and/or validating electrochemical models, equivalent electrical circuit models, statistical models, machine learning models, or artificial intelligence methods to estimate the SOC, current-voltage response, SOH, remaining useful life, and so on. It follows that they can be reused by any researcher trying to create new methods and models in this area.

## 2 Data Description

**Fig. 1:**
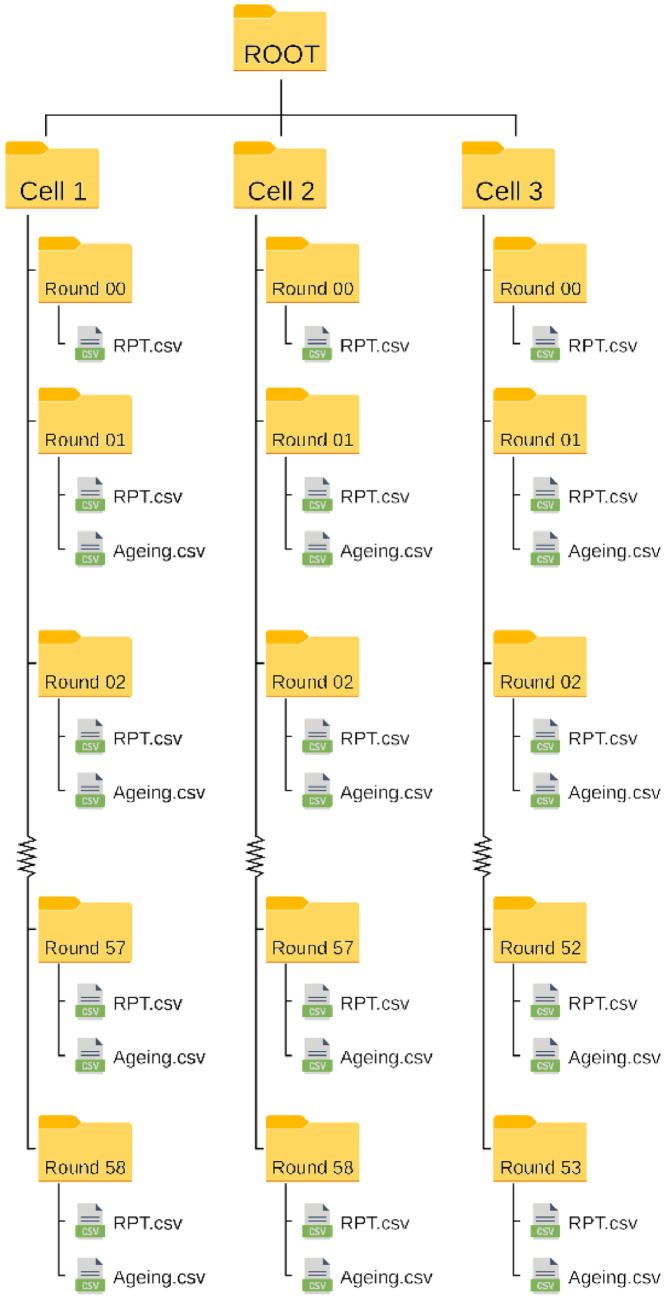
The folder structure of the dataset.

This dataset contains second-by-second measurement information of three Li-ion battery cells, named Cell 1-3, which were aged using a realistic forklift loading profile, parts of which were presented in [3]. The general structure of the dataset can be seen in Fig. 1. It contains three main folders (one for each cell), each folder contains a sub-folder for every round of ageing the cell was subjected to during the experiment. That is, the folders ‘Cell1’, ‘Cell2’, and ‘Cell3’ contain 59, 59, and 54 sub-folders, respectively, using the naming scheme ‘Round00’-‘Round58’.

Each sub-folder contains (up to) two files, named ‘Ageing’ and ‘RPT’. The ‘RPT’ files contain the results of an RPT, described in the section on experimental design, materials, and methods, while the ‘Ageing’ files contain the actual battery response to the applied two-week realistic forklift load profile. Furthermore, all folders ending in ‘00’ contain a single ‘RPT’ file, in this context representing the battery at the beginning of its life, i.e., before any ageing tests were performed. It can also be used to account for any difference in the manufacturing of the cells.

An overview of the measurement information found in the ‘RPT’, and ‘Ageing’ files can be found in Table 1. It should be noted that the files contain the same measurement information, except for ‘Ageing’ having a column called ‘Part’ which is not found in the RPT files. This column is included in the ‘Ageing’ files due to a size limitation, in the software of the battery test station, which was used to apply the realistic forklift load profile; thus, the load profile had to be separated into two parts. While the parts have been collected into a single ‘.csv’ file, it is worth noting that measured time resets from one part to the next, and that some parts have been lost; a complete list of missing parts can be found in Table 2, which also contains information on missing RPT's. Furthermore, if a part, or an RPT, is missing it does not mean that the cell was not subjected to it during ageing, only that it has since been lost. It is worth noting that this loss of information can have implication for the way the data needs to be handled and modelled. It follows that any data cleaning, feature extraction, or modelling, would need to be either agnostic to the missing data, or need to account for it; this could be done either through continuous time models, predictor-corrector schemes, or data imputation and interpolation.

**Table 1:** An overview of the measured variables found in the 'Ageing' and 'RPT' files.

Name	Description
Cell	The cell id; a number from 1 to 3.
Round	The round of ageing; a number from 0 to 58.
Part (not found in the RPTs)	The part of the ageing profile; a number from 1 to 2.
Time	Time measured in seconds [s].
Current	Current measured in Amperes [A].
Voltage	Voltage measured in Volts [V].
Energy	Energy measured in Watt-hours [Wh].
Temperature	Temperature measured in Celsius [*°*C].

**Table 2:** A complete list of missing ageing parts and RPT's for cells 1, 2, and 3.

Round	Cell 1	Cell 2	Cell 3
20			2
22	2	2	
24		1	
25	2	1	RPT
26			1
28	RPT		1, 2, and RPT
29	1, 2, and RPT	1, 2, and RPT	1, 2 and RPT
30	1	1	1 and 2
33			2 and RPT
34	2	2	1 and 2
37	RPT	RPT	
39	2	2	2
42	2	2	
44			2
46	1		
48			1 and 2
51	RPT	RPT	
52	1	1	
56	1 and 2	1 and 2	

## 3 Experimental Design, Materials and Methods

The Li-ion batteries used in this study are prismatic cells using lithium iron phosphate (LFP) and graphite as cathode and anode active materials, respectively. The most important electrical and thermal parameters of the cells are summarized in Table 3. All the ageing tests and RPTs were performed using a Digatron battery test station MCT 50-06-24. Furthermore, during all the tests, the three battery cells were placed inside a Memmert ICP-600 climatic chambers in order to ensure stable and reliable temperature.

**Table 3:** Main parameters of the tested Lithium-ion battery cell.

Parameter	Value
Format	Prismatic
Chemistry	LFP/C
Nominal capacity	180 Ah
Nominal voltage	3.2 V
Maximum voltage	3.65 V
Minimum voltage	2.5 V
Maximum continuous discharging current	180 A (between -20°C and 55°C)
Maximum continuous charging current	18 A (between 0°C and 5°C) 90 A (between 5°C and 10°C) 180 A (between 10°C and 45°C)

**Fig. 2:**
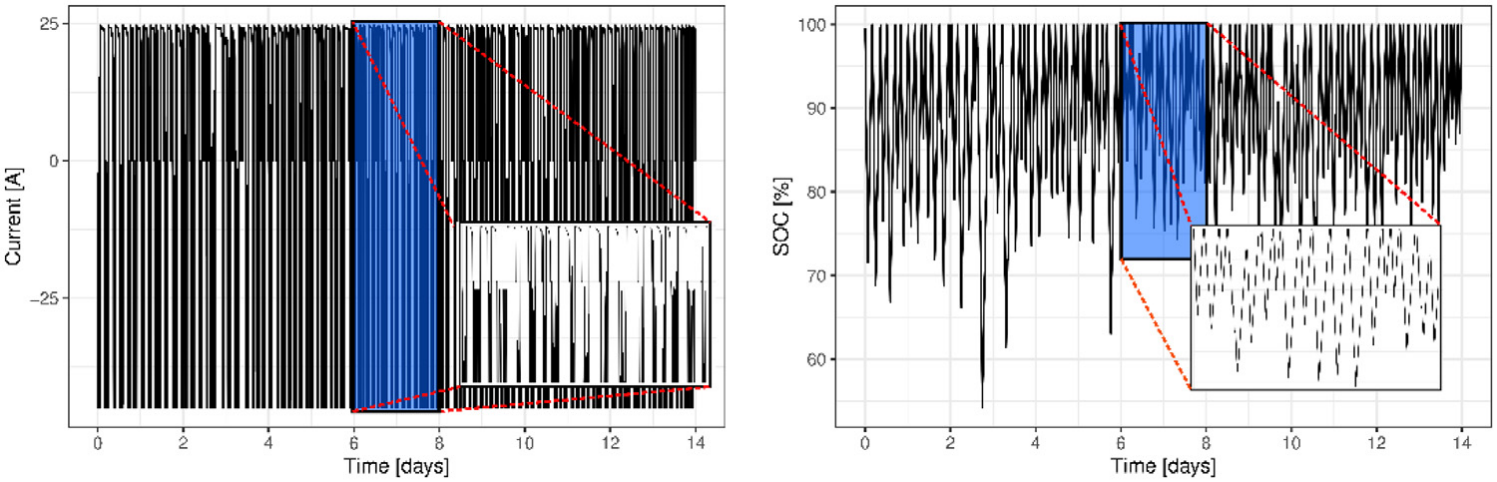
The current and SOC of the realistic forklift load profile used to dynamically age the battery cells.

The realistic forklift load profile, with a length of approximately two weeks, was distilled from a four-month forklift field operation [3]. To accelerate the ageing of the battery cells, all the idling periods found in the forklift field operation were removed in the realistic forklift load profile. Furthermore, to match the specifications of the battery test stations, the charging/discharging current during the ageing with the realistic forklift load profile was limited to 50 A. The SOC profile of the battery cells aged using the realistic forklift load profile was identical to the SOC profile of the forklift field-operated batteries (except the idling periods, which were removed from the earlier). The current and SOC of this two-week profile can be seen in Fig. 2. The temperatures used to age the cells were 45°C for Cell 1, 40°C for Cell 2 and, and 35°C for Cell 3. Note that a subset of this data (i..e., less than half for the cells subjected to the forklift operations profile) was used in a previous manuscript [3], which also includes three additional cells subjected to calendar ageing (i.e. they were charged to 90% SOC and stored at different temperatures for a month). These three cells were excluded from this manuscript for two reasons (1) they were not subjected to the forklift operation profile, and (2) their results were in line with the literature making the data obsolete.

**Fig. 3:**
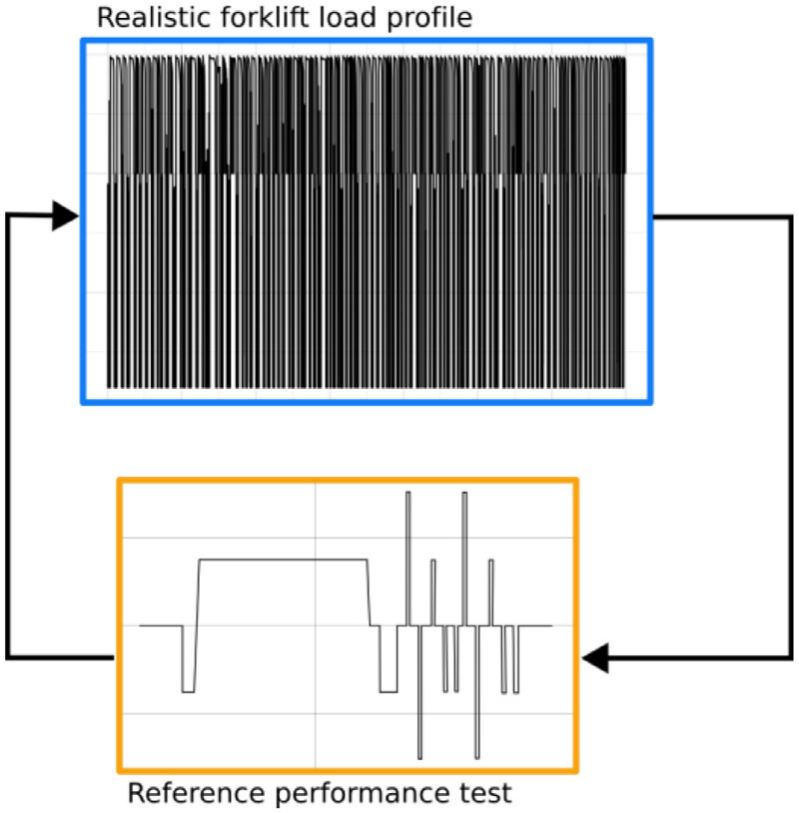
A round of ageing consisting of subjecting the battery cell to the realistic forklift load profile, followed by an RPT.

A single round of battery cell ageing is comprised of two steps: (1) the ageing itself (using the realistic forklift load profile illustrated in Fig. 2, and (2) an RPT. These two steps were repeated 58 times for Cell 1 and Cell 2, and 53 times for Cell 3, as depicted in Fig. 3.

**Fig. 4:**
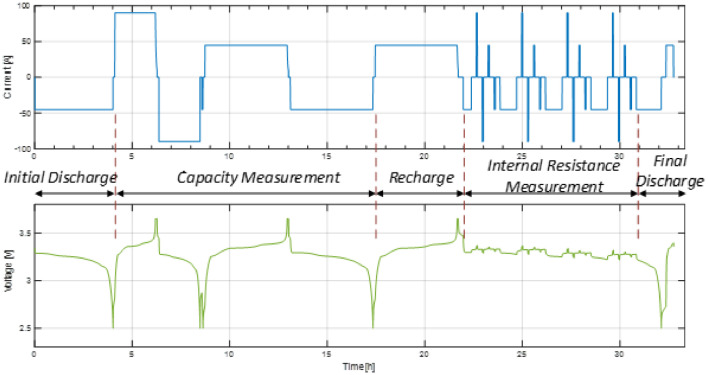
Battery cell current (top) and voltage (bottom) during an RPT (where a positive current denotes battery charging, a negative current denotes battery discharging, while zero current denotes a battery in standby).

The RPT was designed, similarly to [1], to measure the capacity and internal resistance of the battery cells and to assess the incremental degradation of these performance parameters during the ageing of the battery cells with the realistic forklift load profile. The current and voltage signals measured on one of the cells during an RPT are illustrated in Fig. 4 highlighting different measurement steps. During the first step, the battery cell is discharged to the minimum voltage (i.e., 2.5 V) with a constant current of 45 A. After a break of one hour, the capacity measurement of the cell is performed with the currents; the battery is charged with a constant current of 90 A until it reaches 3.65 V and then with constant voltage until the current drops to 9 A, while after a break of one hour, the battery is discharged with a constant current of 90 A until it reaches 2.5 V. The charging and discharging capacity measurement is then repeated for a current of 45 A. Then, prior to the internal resistance measurement, the battery cell is fully recharged with 45 A using a constant current – constant voltage protocol. The internal resistance of the battery cell was measured, following the procedure in [2], at four SOCs (i.e., 90%, 70%, 50%, and 30%) during both charging and discharging by applying 20 seconds currents of 90 A and 45 A, respectively; before each internal resistance measurement, a 15-minute break was applied. Furthermore, a current of 45 A was used to discharge the battery to the SOCs. Finally, in the last RPT step, the battery is discharged with 45 A to the minimum voltage (i.e., 2.5 V) and after a break of 15 minutes, the battery is charged to 90% SOC with a current of 45 A. The RPT was always performed at 25°C.

**Fig. 5:**
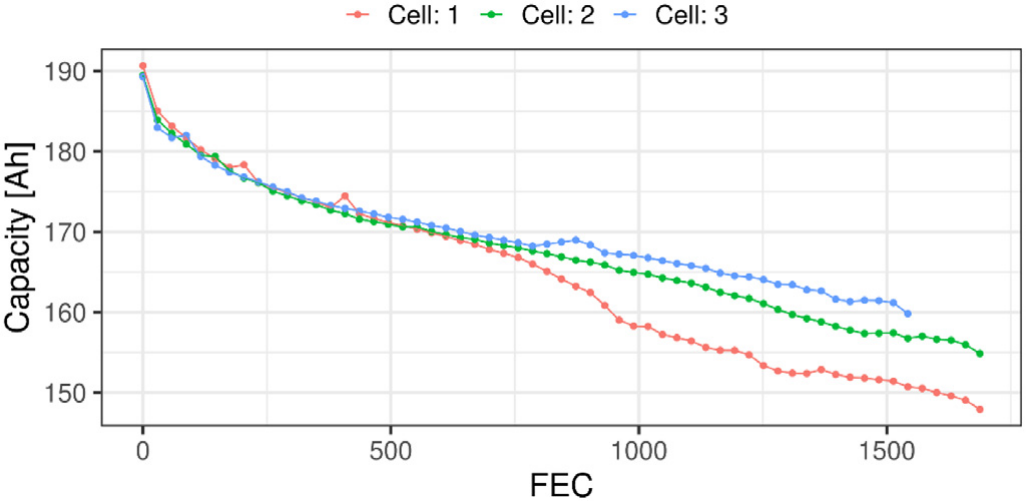
The full capacity against the FEC, extracted from the RPT's at C/4 (i.e., 45 A) and 25°C for every round of ageing and all three cells.

Using the RPT performed at the end of each round of ageing, the full capacity and internal resistance can be extracted for each of the cells. The capacity degradation behaviour of the three cells, based on capacity measurements performed at C/4 (i.e., 45 A), can be seen in Fig. 5. The figure shows the measured capacity against full equivalence cycles (FEC). The FEC at time t can be found as:

$$FEC\left(t\right)=\frac{1}{2\;\cdot Q_{norm}\;\cdot \;3600\;}\int_{0}^{t}\left|\;I\left(\tau \right)\;\right|\;d\tau ,$$

where I(t) is the current at time t, and $Q_{norm}$ is the nominal capacity of the cell found in Table 3. In this data each round of ageing corresponds to roughly 29 FEC.

## Limitations

There are three limitations of the dataset: (1) some rounds of ageing and/or RPTs were lost due to issues with the experimental set-up, (2) the operations profile is exactly the same in each round of ageing, and (3) calendar and cycling ageing were de-coupled at the design level, i.e. interactions between the two cannot be studied using this dataset.

## Ethics Statement

The authors have read and followed the ethical requirements for the publication of Data in Brief, and hereby confirm that the work did not involve data collected from human subjects, animal experiments, or social media platforms.

## CRediT authorship contribution statement

**Søren B. Vilsen:** Investigation, Software, Visualization, Writing – review & editing. **Daniel-Ioan Stroe:** Conceptualization, Data curation, Methodology, Visualization, Writing – review & editing.

## Data Availability

Lithium-ion battery degradation dataset based on a realistic forklift operation profile (Original data) (Mendeley Data)
